# The influence of different anticoagulants and sample preparation methods on measurement of mCD14 on bovine monocytes and polymorphonuclear neutrophil leukocytes

**DOI:** 10.1186/1756-0500-5-93

**Published:** 2012-02-14

**Authors:** Eveline M Ibeagha-Awemu, Aloysius E Ibeagha, Xin Zhao

**Affiliations:** 1 Department of Animal Science, McGill University, Ste-Anne-de-Bellevue, Quebec H9X 3V9, Canada; 2 Dairy and Swine Research and Development Centre, Agriculture and Agri-Food Canada, Sherbrooke, Quebec J1M 0C8, Canada

**Keywords:** Membrane CD14, Monocytes, Polymorphonuclear neutrophil leukocytes, Sodium heparin, Ethylenediaminetetraacetic acid, Sodium citrate, Flow cytometry, Holstein cows

## Abstract

**Background:**

Membrane-CD14 (mCD14) is expressed on the surface of monocytes, macrophages and polymorphonuclear neutrophil leukocytes (PMN). mCD14 acts as a co-receptor along with Toll like receptor 4 (TLR 4) and MD-2 for the detection of lipopolysaccharide (LPS). However, studies using different sample preparation methods and anticoagulants have reported different levels of mCD14 on the surface of monocytes and neutrophils. In this study, the influence of various anticoagulants and processing methods on measurement of mCD14 on monocytes and neutrophils was examined.

**Results:**

Whole blood samples were collected in vacutainer tubes containing either sodium heparin (HEPARIN), ethylenediaminetetraacetic acid (EDTA) or sodium citrate (CITRATE). mCD14 on neutrophils and monocytes in whole blood samples or isolated cells was measured by the method of flow cytometry using fluorescein isothiocyanate (FITC)-labeled monoclonal antibody. There was a significant difference (*p *< 0.05) in the mean channel fluorescence intensity (MFI) of mCD14 on neutrophils in whole blood samples anticoagulated with HEPARIN (MFI = 64.77) in comparison with those in whole blood samples anticoagulated with either EDTA (MFI = 38.25) or CITRATE (MFI = 43.7). The MFI of mCD14 on monocytes in whole blood samples anticoagulted with HEPARIN (MFI = 206.90) was significantly higher than the MFI in whole blood samples anticoagulated with EDTA (MFI = 149.37) but similar to that with CITRATE (MFI = 162.55). There was no significant difference in the percentage of whole blood neutrophils or monocytes expressing mCD14 irrespective of type of anticoagulant used. However, MFI of mCD14 on monocytes was about 3.2-folds (HEPARIN), 3.9-folds (EDTA) or 3.7 folds (CITRATE) higher than those on neutrophils. Furthermore, there was no significant difference in mCD14 levels between unprocessed whole blood monocytes and monocytes in peripheral blood mononuclear cell preparation. Conversely, a highly significant difference was observed in mCD14 between unprocessed whole blood neutrophils and isolated neutrophils (*p *< 0.05).

**Conclusion:**

From these results, it is suggested that sodium heparin should be the preferred anticoagulant for use in the reliable quantification of the surface expression of mCD14. Furthermore, measurement of mCD14 is best carried out in whole blood samples, both for neutrophils and monocytes.

## Background

Membrane CD14 (mCD14) is a membrane-associated glycosylphosphatidylinositol (GPI)-linked receptor protein [1]. It is constitutively expressed on the surface of various cells, including monocytes, macrophages and neutrophils. CD14 is a high affinity receptor protein for the complexes of bacterial lipopolysaccharide (LPS) and LPS-binding protein [2]. In addition to its membrane-expressed form, CD14 can also be found in blood and milk as soluble CD14 (sCD14) [3,4]. Both membrane and soluble forms of CD14 bind to Gram-negative bacteria [5]. It has also been demonstrated that CD14 is capable of binding with other bacterial and yeast cell wall components [6] and because of this multiple identification, CD14 is referred to as a "pattern recognition receptor" [7]. In addition, CD14 works in combination with Toll-like receptors, members of the interleukin family, for the transmission of intracellular signals. For example, CD14 acts as a co-receptor along with Toll like receptor 4 (TLR 4) and MD-2 for the detection of LPS [8,9].

Both neutrophils and monocytes/macrophages are important to combat invading bacteria. At the site of infection, phagocytes (predominantly granulocytes and monocytes/macrophages) destroy Gram-negative bacteria by phagocytosis, mostly through the LPS receptor, CD14 on the cell surface [10]. However, there are some discrepancies in the reported levels of mCD14 expression on the surface of monocytes and neutrophils (Table 1). These reports employed different sample preparation methods and blood anticoagulants [11-14] which could have affected the results. Anticoagulation is achieved either by the binding of calcium ions (EDTA and citrate) or by the inhibition of thrombin (heparin). Heparin is the preferred anticoagulant for most clinical chemistry analyses and for the measurement of some trace elements, ammonia, blood pH and blood gas analysis [15]. EDTA is particularly useful for hematological examination. On the other hand, sodium citrate solution is widely used for coagulation studies because the effect is easily reversible by the addition of Ca + 2 (calcium ions). However, these commonly used anticoagulants have been reported to have varying effects on blood components [16,17]. Therefore, blood handling and the choice of anticoagulant may have an effect on the quality of data and potentially result in analytical bias.

**Table 1:** Effects of different isolation methods on the percentage of PMN or monocytes expressing mCD14 in different species

Source	Percentage of PMN expressing mCD14	Percentage of monocytes expressing mCD14	Method	Authors
Human	ND	90	Whole blood	[18]
Goat	47.2	2.60	Isolated cells	[19]
Ilama	45.23	13.58	Isolated cells	[19]
Rabbit	34.63	4.24	Whole blood	[19]
Bovine	40-95	60-95	Whole blood	[20]
Bovine	35.6	ND	Isolated cells	[21]

ND: Not determined

Little is known about the influence of sample handling and the various types of commonly used anticoagulants in relation to the quantification of mCD14 on monocytes and neutrophils in bovine.

The aim of this study therefore was to investigate the effects of anticoagulants and cell preparation procedures on measurement of mCD14 on monocytes and neutrophils.

## Methods

### Animals

Eighteen mid- to late-lactating Holstein cows (220 ± 60 d of lactation) were randomly selected for this study. The cows were in their first to third parities. The monthly assessment of farm records (individual cows) indicated that the animals were in general good health. The cows were individually housed, during the entire experimental period in identical stalls with sawdust as bedding, had ad libitum access to drinking water and were fed the standard farm rations for their physiological stages. This study was approved by the McGill University Animal Care Committee.

### Blood sampling

Blood was collected aseptically from the caudal vein by venipuncture into one of three vacutainer tubes containing either sodium heparin (HEPARIN), sodium citrate (CITRATE) or ethylenediaminetetraacetic acid (EDTA) (Becton Dickinson, Franklin Lakes, NJ, USA). These tubes were immediately placed on ice and samples were prepared or analyzed within 45 minutes of collection.

### Isolation of polymorphonuclear neutrophil leukocytes (PMN)

Eight millilitres of whole blood was aseptically collected in vacutainer tubes containing either HEPARIN, CITRATE or EDTA and immediately placed on ice. The PMN was isolated from whole blood according to an established protocol in our laboratory [22]. The cell pellet was washed twice with 1 × DPBS (Dulbecco's phosphate buffered saline) by subsequent centrifugation at 250 × *g *for 5 minutes at 4°C. The cell pellet was resuspended in 1 mL of 1 × DPBS and kept on ice. Live cells were counted by using a haemacytometer and the trypan blue exclusion method. The cells showed 95% viability. Cells were adjusted to a concentration of 1.0 × 10^6 ^viable PMN/mL.

### Isolation of peripheral blood mononuclear cells (PBMC)

Bovine peripheral blood mononuclear cells (PBMC) were isolated from whole blood preparations of healthy cows by the use of density gradient centrifugation over Ficoll-Paque (density 1.077; Amersham Biosciences, NJ, USA). PBMC were isolated with some slight modifications as described by Weiss et al. [23]. All solutions and equipment coming into contact with the cells were sterilized and the whole process was performed under sterile conditions. Briefly, 7 mL of whole blood samples were diluted 1:1 (v/v) with 7 mL of 1 × DPBS. The resulting solution was carefully overlayed on 20 mL of Ficoll-Paque solution. The tube was centrifuged at 400 × *g *for 40 minutes at room temperature to separate mononuclear cells from other fractions. Cells from the interface (PBMC) were removed and placed in a 50 mL clean sterilized falcon tube. Tubes were then filled with ice-cold 1 × DPBS and centrifuged at 400 × *g *at 4°C. The resulting pellet from the centrifugation was resuspended in 1 × DPBS and washed twice by centrifugation for 8 minutes at 400 × *g*. After these wash steps, the pellet was resuspended in 1 × DPBS and adjusted to a concentration of 1.0 × 10^6 ^cells/mL.

Further isolation of monocytes from PBMC could be achieved by adhesion or positive and negative immunoselection but these procedures are laborious and time-consuming. Thus, further isolation was not carried out in this study to avoid additional effect of the lengthy procedure.

### Multiparametric flow cytometry

Flow cytometry was used to study the influence of the different anticoagulants on mCD14 on monocytes and neutrophils in healthy cows. For the assay using whole blood samples, 200 μL of whole blood was placed in a 12 × 75 mm flow cytometric (FCM) tube and incubated with 10 μL of fluorescein isothiocyanate (FITC)-labeled mouse anti human CD14 antibody (ABD Serotec Inc. Raleigh, NC, USA). This was mixed thoroughly and incubated at room temperature on an orbitron rotator (Boekel Ind. Inc., PA, USA) for 30 minutes. Lysis of erythrocytes was performed by adding 2 ml of a lysing solution (0.87% Tris buffered ammonium chloride solution (NH_4_CL)) to the mixture. This was mixed gently and incubated on an orbitron rotator at room temperature and centrifuged at 250 × *g *for five minutes at 4°C. The supernatant was aspirated leaving approximately 400 μL of cells in the FCM tube. This was washed with 3 mL of ice-cold 1 × DPBS, pH 7.2 (Invitrogen) by centrifuging at 250 × *g *for 5 minutes at 4°C. The supernatant was aspirated as described above and the cells resuspended in 400 μL of ice-cold 1 × DPBS. The cells were then washed by centrifugation and fixed with 2% DPBS-buffered paraformaldehyde and analysed within 60 minutes with a BD FACSCalibur (Becton Dickinson Immunocytometry Systems, San José, CA, USA). The FACSCalibur was equipped with an air-cooled argon ion laser (488 nm, 15 mW) and a diode laser (635 nm, 9 mW). This standard instrument is equipped with two light scatter detectors that measure forward and side scatter and four fluorescence detectors that detect appropriately filtered light at 525 nm.

Excitation of samples was at 488 nm, with FITC fluorescence measured at 525 nm ± 10 nm. Acquisition was stopped when 30,000 gated events per sample were collected. Gating of monocytes and PMN was based on forward side scatter and side scatter dot plots. All parameters were recorded with the logarithmic amplifications. The data analysis was performed using Cell-Quest Software (Becton Dickinson). List mode flow cytometric data from 30,000 events were stored and processed with the Windows Multiple Document Interface for Flow Cytometry (WinMDI) software version 2.8 (Joseph Trotter, The Scripps Research Institute, http://facs.scripps.edu/software.html). The software constructs a histogram of fluorescence distribution and the relative mean fluorescence intensity (MFI) was obtained and expressed as an index of surface expression. The control was a FITC-conjugated immunoglobulin of the relevant isotype (ABD Serotec Inc., Raleigh, NC, USA).

### Multiparametric flow cytometry for isolated cells

Isolated cells, about 1.0 × 10^6 ^cells/mL, were similarly analyzed as detailed out in the previous section. Isolated cells were placed in a 12 × 75 mm FCM tube and incubated with 10 μL of fluorescein isothiocyanate (FITC)-labeled mouse anti human CD14 antibody (ABD Serotec Inc. Raleigh, NC, USA). This was mixed thoroughly and incubated at room temperature on an orbitron rotator (Boekel Ind. Inc., PA, USA) for 30 minutes. Cells were later washed twice with 3 mL of 1 × DPBS by centrifuging at 250 × *g *for five minutes at 4°C. The cells were then washed by centrifugation with 1 × DPBS and fixed with 2% DPBS-buffered paraformaldehyde and analysed within 60 minutes with a BD FACSCalibur (Becton Dickinson Immunocytometry Systems, San José, CA, USA). Monocyte and neutrophil populations were identified by their light scatter properties as the side scatter and the forward scatter on dot plots.

### Statistical analysis

Flow cytometric data (percent fluorescence and MFI) were analysed as a one way ANOVA using the MIXED procedures of SAS version 6.1 program [24]. Treatment means were separated using the least square means option of SAS. Differences between treatment means were tested using the Scheffe's Multiple Comparison test. Statistical significance was declared at *P ≤ 0.05*.

$$\text{Statistical}\;\text{model}\;\text{used}:\;{\text{Y}}_{\text{ij}}=\mu +{\text{anticoagulant}}_{\text{i}}+{\text{e}}_{\text{ij}}$$

## Results

### Effects of different anticoagulants on mCD14 on neutrophils

**Figure 1:**
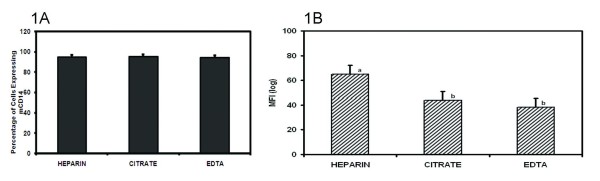
**Impact of different blood anticoagulants on the surface expression of CD14 on bovine neutrophils**. Whole blood samples obtained from 18 Holstein cows were anticoagulated with: sodium heparin (HEPARIN), sodium citrate (CITRATE) and ethylenediaminetetraacetic acid (EDTA). The percentage of PMN expressing mCD14 (1A) and the mean channel fluorescence intensity (MFI) (1B) was measured. Results for each treatment are the mean from 6 cows. Treatment means with different superscripts are significantly different (*p *< 0.05) using Scheffe's multiple comparison test.

The effect of different commonly used anticoagulants on the surface expression of bovine mCD14 on neutrophils was first investigated on whole blood samples. There was no significant difference in the percentage of PMN expressing mCD14 among the different anticoagulants used with a mean of 94.85%, 94.42%, and 94.70% for HEPARIN, CITRATE and EDTA respectively (Figure 1A). However, when the density of mCD14 on neutrophils was compared, HEPARIN, with a MFI of 64.77 was significantly different (*p *< 0.05) from CITRATE (43.70) and EDTA (38.25) anticoagulated blood samples (Figure 1B).

### Effects of using different anticoagulants on the expression of mCD14 on monocytes

**Figure 2:**
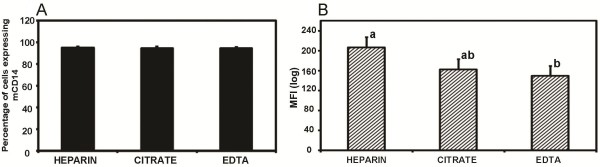
**Impact of different blood anticoagulants on the surface expression of mCD14 on bovine monocytes**. Whole blood samples obtained from 18 Holstein cows were anticoagulated with: sodium heparin (HEPARIN), sodium citrate (CITRATE) and ethylenediaminetetraacetic acid (EDTA). The percentage of monocytes expressing mCD14 (2A) and the mean channel fluorescence intensity (MFI) (2B) was measured. Results for each treatment are the mean from 6 cows. Treatment means with different superscripts are significantly different using Scheffe's multiple comparison tests.

Similarly, the effect of different commonly used anticoagulants on the surface expression of bovine mCD14 on monocytes was investigated in whole blood samples. There was no significant difference in the percentages of monocytes expressing mCD14 among the three groups (Figure 2A). However, when the MFI was compared, the HEPARIN group with a MFI of 206.90 was significantly higher than the EDTA group with a MFI of 149.37 but was not significantly different from the CITRATE group with a MFI of 162.55 (Figure 2B). In addition, there was no significant difference between CITRATE and EDTA anticoagulated blood.

### Effects of isolation procedures on the expression of mCD14 on neutrophils

**Figure 3:**
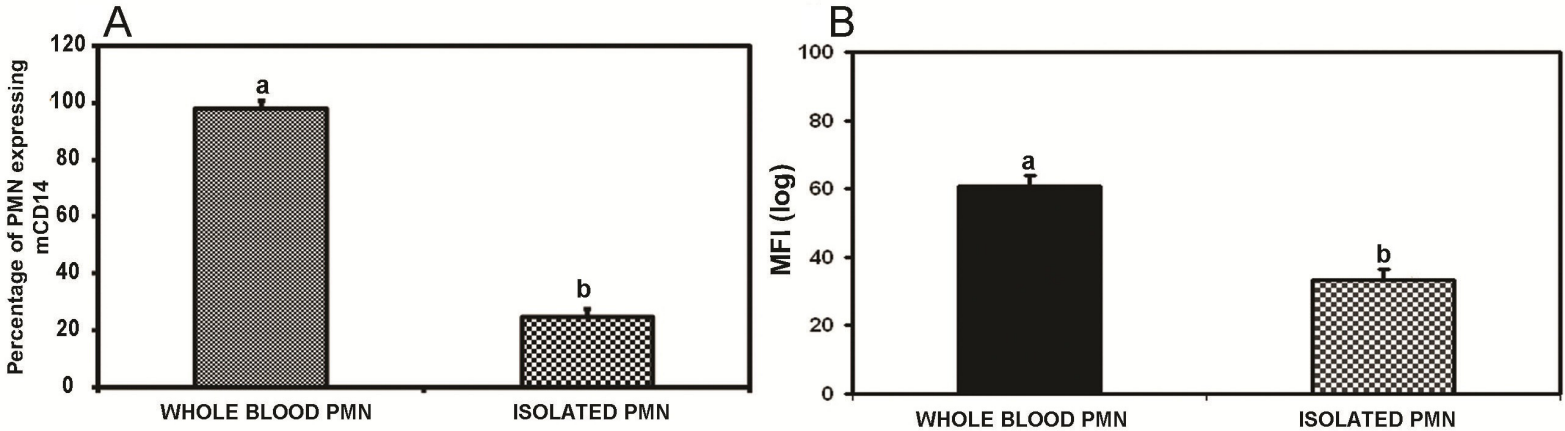
**Effect of the isolation procedure on the surface expression of CD14 on neutrophils**. Whole blood or isolated neutrophil was stained with CD14 mAb. Blood was anticoagulated with HEPARIN. Using flow cytometry, the percentage of PMN expressing mCD14 (3A) and the mean channel fluorescence intensity (MFI) (3B) was measured. Results for each treatment are the mean from 5 cows. Treatment means with different letters are significantly different (*p *< 0.05) using Scheffe's multiple comparison tests.

In order to determine whether sample preparation affected the quantification of mCD14, mCD14 on whole blood or isolated leukocytes was determined. Samples used were anticoagulated with HEPARIN only. There was a significant difference in the percentage of PMN expressing mCD14 (Figure 3A) which drastically reduced from 97.97% observed in whole blood neutrophils to 24.82% in isolated neutrophils. Furthermore, whole blood neutrophils recorded a higher number of mCD14 molecules as measured by the MFI of 60.70 on their surfaces, which was significantly different from isolated neutrophils, 33.27 (Figure 3B).

### Effects of isolation procedures on the expression of mCD14 on monocytes

**Figure 4:**
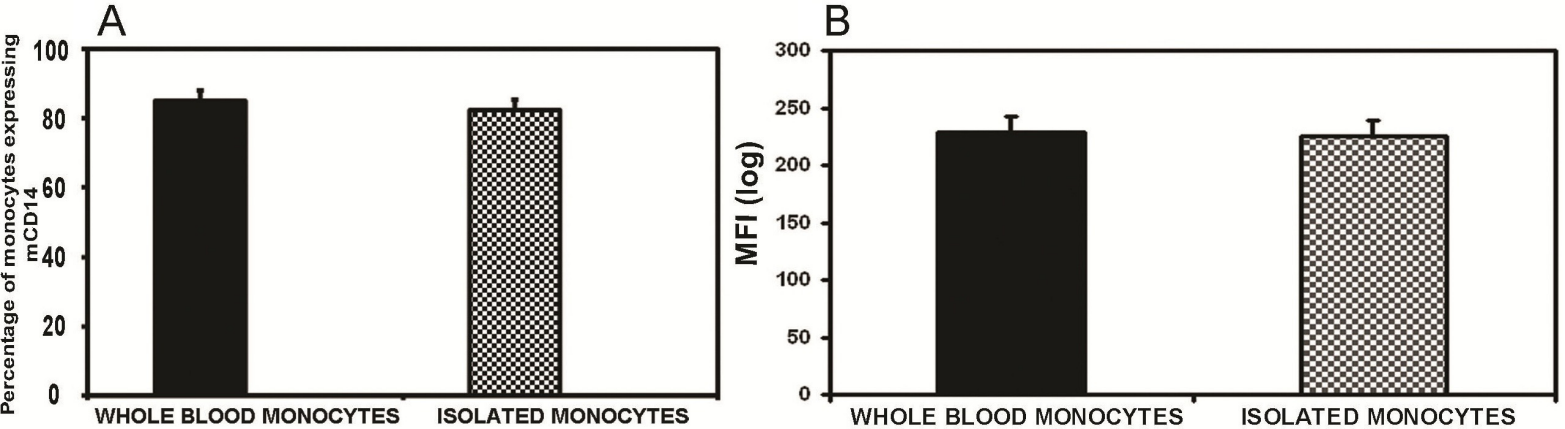
**Effect of the isolation procedure on the surface expression of mCD14 on monocytes**. Whole blood monocytes or isolated PBMC was stained with CD14 mAb. Blood was anticoagulated with HEPARIN. The percentage of monocytes expressing mCD14 (4A) and the mean channel fluorescence intensity (MFI) (4B) was measured. 30,000 cells were gated and analysed for each event. Results for each treatment are the mean from 5 cows.

In contrast to neutrophils, the percentages of whole blood monocytes (84.89%) expressing mCD14 on their surfaces (anticoagulated with HEPARIN) and monocytes from isolated PBMC (82.34%) (blood was anticoagulated with HEPARIN) were not significantly different (Figure 4A). Likewise, the MFI of whole blood monocytes (228.48) did not change significantly when compared to monocytes in isolated PBMC (225.15) (Figure 4B).

## Discussion

Blood collection and sometimes isolation of immune cells are necessary for the measurement of blood immune parameters ex-vivo. Anticoagulatants prevent blood clotting before analysis of samples. In this study, flow cytometry was used to determine mCD14 expression on whole blood neutrophils and monocytes as well as on isolated monocytes and neutrophils from blood samples collected into tubes containing different anticoagulants. Our study establishes that both anticoagulant and processing method has an effect on the levels of mCD14 on bovine cells.

In this study, we show that sodium heparin is the preferred choice of anticoagulant to use in the quantification of mCD14 on neutrophils and monocytes. Although the number of cells expressing mCD14, whether monocytes or neutrophils, was the same irrespective of type of anticoagulant in our study, the density of mCD14 was significantly higher on cells from samples anticoagulated in heparin than in CITRATE and EDTA (Figures 1B and 2B). Our results are similar to the findings of Harding et al. [25] who reported a significantly higher platelet-monocyte aggregation level in blood anticoagulated with heparin as compared to D-Phenylalanine-L-prolyl-L-arginine chloromethyl ketone (PPACK), sodium citrate or EDTA. Similarly, Walter et al. [26] observed that levels of bioactive TGF-beta in blood collected into heparin but not EDTA tubes remained stable for longer periods (up to 18 h), even when kept at room temperature. Therefore, they recommended that heparin should be the choice anticoagulant when reliable estimates of TFG-beta are needed. Heparin is a naturally-occurring anticoagulant produced by basophils and mast cells of mammals. It prevents the formation of clots and extension of existing clots within blood through the inhibition of thrombin and is generally recommended as the suitable anticoagulant for plasma biochemical measurements [15]. Heparin has the advantage over EDTA of not affecting the levels of most ions in blood. However, heparin must be used with caution because it has been shown that the levels of ionized calcium may be decreased if the concentration of heparin in the blood specimen is too high [27]. The different effects of anticoagulant and isolation procedure on levels of mCD14 could be mostly due to shedding of mCD14 from cell surface. It has been shown that under certain conditions monocytes/macrophages can remove cell-bound IgG without destroying the opsonised cell [28]. A recent study [29] showed that this mechanism accounts for a phenomenon called 'shaving', where monocytes can remove anti-CD20 antibodies together with CD20 from the surface of antibody-coated target cells. This process occurs through an endocytic reaction called trogocytosis that depends on Fcc receptor I expression on the acceptor cell [29]. When this happens, the shaved target cells are viable but have reduced CD20 expression [30]. Furthermore, Pedersen et al. [31] demonstrated that a monocyte-mediated shaving reaction can lead to complete loss of most anti-CD20 antibodies from the surface of B cells. The effect of shedding observed in our study could be explained by this mechanism of shaving. Our results showed a more pronounced effect of EDTA and CITRATE on the shedding of mCD14 in comparison with heparin. Both EDTA and CITRATE are calcium chelators and might be involved in other divalent cation-dependent interactions which may interfere with other processes thus leading to the much lower levels of mCD14 on monocytes and neutrophils [12]. Sarma et al. [32] have demonstrated that cation chelation with EDTA markedly reduces platelet-leukocyte interactions in vitro. Also, a reduction in platelet-neutrophil binding with EDTA and citrate has been demonstrated [25,33]. These could further explain the significantly lower density of mCD14 on the surface of monocytes and neutrophils in blood anticoagulated with the calcium chelators, EDTA and CITRATE.

By comparing the mCD14 levels on leukocytes in whole blood and after Ficoll-Paque density gradient isolation, we showed that the isolation procedure had no effect on the number of monocytes expressing mCD14 but had a drastic effect on the number of neutrophils expressing mCD14. Human monocytes expressed about 99,500-134,600 CD14 molecules on their surfaces and human neutrophils about 1,900-4,400 [11]. In a recent study, these differences were confirmed in bovine cells by Ibeagha-Awemu et al. [20] who recorded monocytes in a zone of higher expression of CD14 on histograms of flow cytometric data while more neutrophils were recorded in a region of lower expression. Their study also demonstrated a higher density of mCD14 on monocytes (MFI = 201.44) than neutrophils (MFI = 34.76) in blood. Thus, the dramatic effect of isolation procedure on neutrophils could be explained by their generally lower numbers and also the lower density of mCD14 molecules on their surfaces as compared to monocytes, which resulted in a pronounced effect of shedding. Other authors have also demonstrated the effect of isolation procedure on neutrophil shedding of mCD14 molecules [21,34].

Our results of similar percentages of PMN or monocytes expressing mCD14 regardless of type of anticoagulant used in whole blood is supported by the findings of Ibeagha-Awemu et al. [20]. The positive results with unprocessed whole blood, apart from saving time and material also allow the measurement of these parameters in a smaller volume of blood. Furthermore, the use of whole blood is simple, fast, requires less handling steps and minimal complex procedures. Conversely, the processes of cell isolation does not only lead to unreliable estimates but the process can be laborious, time consuming, require complex steps and larger starting material and can also be costly.

## Conclusion

In summary, we propose that reliable mCD14 measurements in blood should be obtained by using freshly obtained whole blood as starting material and heparin as blood anticoagulant. Our findings have provided important methodological information that should be considered in the reliable estimation of the mCD14 protein, a crucial molecule that contributes to host innate recognition of bacterial cell wall components.

## Competing interests

The authors declare that they have no competing interests.

## Authors' contributions

XZ conceived the study and supervised the experiment; AEI and EMI-A carried out the experiments, analyzed data and wrote the manuscript. All authors read and approved the final manuscript.
